# Xanthomonas citri pv. eucalyptorum strain 4866-2_S43 (formerly X. axonopodis pv. eucalyptorum): the causal agent of bacterial leaf blight on eucalypts recovered in Argentina

**DOI:** 10.1099/acmi.0.000827.v3

**Published:** 2024-10-07

**Authors:** German Matias Traglia, Mousami Poudel, Samuel Miño, Blanca Isabel Canteros, G. V. Minsavage, Anuj Sharma, Erica M. Goss, Jeffrey B. Jones, Alberto Gochez

**Affiliations:** 1 Unidad de Genómica y Bioinformática, Departamento de Ciencias Biológicas, CENUR Litoral Norte, Universidad de la República, Salto, Uruguay; 2 Department of Plant Pathology, University of Florida, Gainesville, FL, USA; 3 Instituto Nacional de Tecnología Agropecuaria (INTA), EEA Cerro Azul, Cerro Azul, Misiones, Argentina; 4 Instituto Nacional de Tecnología Agropecuaria (INTA), EEA Bella Vista, Bella Vista, Corrientes, Argentina

**Keywords:** ANI, eucalyptus, genome assembly, *Xanthomonas*

## Abstract

We report the draft genome assembly of strain 4866-2_S43 isolated from a eucalyptus lesion in Argentina and what until recently was caused by *Xanthomonas citri* pv. *eucalyptorum* (*Xce*). The genome size is 5 188 607 bp, with a G+C content of 64.66%. Comparative analysis reveals that the closest relative of strain 4866-2_S43 is *Xce* LPF 602, isolated in Brazil. Comparison of the whole genome sequences revealed an average nucleotide identity (ANI) of 99.96% between the two strains. ANIs were determined between the whole genome sequence of strain 4866-2_S43 and the genomes of all currently validated *Xanthomonas* spp. These results revealed that strain 4866-2_S43 shared >95% similarity with *X. citri* pv. *citri* and *X. citri* pv. *phaseoli*, and <95% with *X. euvesicatoria* pv. *alfalfae*, *X. euvesicatoria* pv. *perforans*, and *X. euvesicatoria* pathovars *euvesicatoria* and *eucalyptii*.

## Data Summary

This whole-genome shotgun project has been deposited at DDBJ/EMBL/GenBank under accession no. JAZGJM000000000. The annotated assembly has been deposited at the U.S. Department of Energy (DOE) Joint Genome Institute (JGI) under accession no. Gp0621574.

## Announcement

Bacterial leaf blight (BLB) constitutes a significant disease affecting eucalyptus trees, particularly in regions characterized by abundant rainfall, both in the open field and nursery settings [1]. Typical symptoms of BLB include leaf distortion and water-soaking associated with lesions, progressing to necrosis that may lead to the formation of shotholes in lesions, ultimately resulting in pronounced defoliation [2]. A comprehensive investigation conducted in 2008, utilizing strains collected from diverse geographical locations, identified *Xanthomonas axonopodis* pv. *eucalyptorum* (*Xae*) [later *X. citri* pv. *eucalyptorum* (*Xce*)] as the causal agent of BLB in *Eucalyptus* spp. within Brazil [2]. More recently, the emergence of a novel pathovar, *X. euvesicatoria* pv. *eucalyptii* (*Xee*), was documented as the causal agent of BLB in *Eucalyptus* spp. [3].

Eighteen *Xce* strains were isolated on nutrient agar (NA) from *Eucalyptus grandis* hybrids in the Experimental Station of INTA Bella Vista (Corrientes province, Argentina) in 2017. All the *Xce* strains elicited a hypersensitive reaction when infiltrated into grapefruit leaves (*Citrus paradisi*). Initially, species identification was performed using standard biochemical methods. The strains were stored at −80 °C in 30% glycerol containing 0.8% nutrient broth (NB, Difco, Becton Dickinson). One of the strains, *Xce* 4866-2_S43, was grown on an NA plate for 24 h and processed for sequencing, and further bioinformatics analyses were done using the methodology as outlined by Subedi *et al*. [4]. The whole genome of *Xae* 4866-2 was sequenced on the Illumina MiSeq, generating a total of 725948 paired ends with a read length of 2×250 bp, resulting in a mean coverage of 8×. Raw sequencing reads were subjected to quality control and adapter trimming using FastQC v0.11.9 (https://www.bioinformatics.babraham.ac.uk/projects/fastqc/) and Trimmomatic v0.38, respectively [5]. Clean reads were then *de novo* assembled using SPAdes v3.15.4 [6], removing contigs smaller than 500 bp to ensure sequencing quality, leaving a total of 209 contigs containing 5  188  607 bp. The maximum and minimum scaffold lengths were 146  011 and 519 bp, respectively. The scaffold N50 and N90 metrics were 51 103 and 14  158 bp, respectively. Furthermore, calculated G+C content was 64.66%. Genome annotation was accomplished by prokka software [7]. A total of 4416 coding sequences and 48 tRNAs were identified in the *Xce* 4866-2_S43 strain genome.

**Fig. 1.**
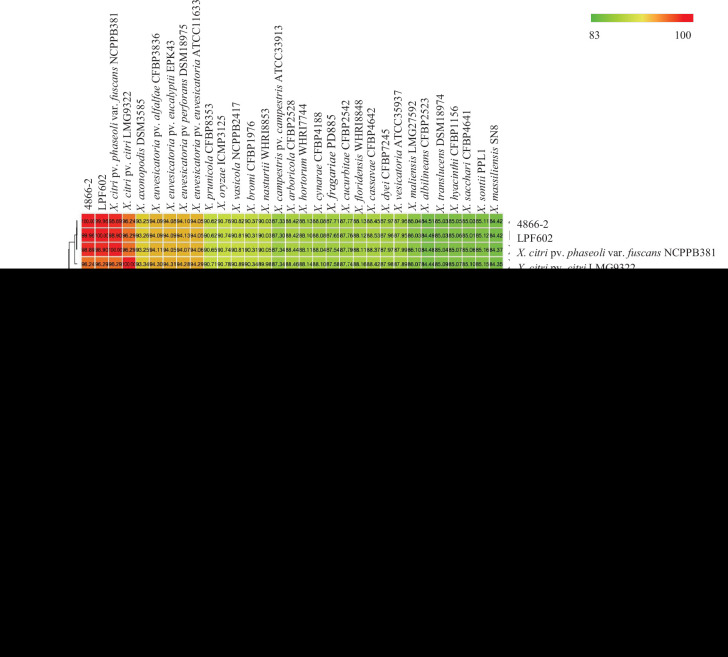
ANI values of strain 4866-2 with the type strains of *Xanthomonas* spp.

Average nucleotide identity (ANI) and *in silico* DNA–DNA hybridization (DDH) analyses of the whole genome of strain *Xce* 4866-2_S43 were performed with the reference genomes of *Xanthomonas* available in the NCBI genome database to determine the correct species identification and estimate the relationship of *Xce* 4866-2_S43 with other *Xanthomonas* spp. ANI and DDH values were determined using ANIb [8,9] and GGDC [10]. The ANI value of *Xce* 4866-2_S43 compared with 30 NCBI reference genomes of the genus *Xanthomonas* and 16 *X*. *axonopodis* genomes was >99% to *Xce* LPF 602 (RWJW00000000.1). Also, DDH comparison with same dataset of genomes showed a DDH value >99% to *Xce* LPF 602 (Fig. 1). Based on our result of multiple genome comparisons through several *in silico* analyses, the strain isolated in Argentina is placed as *Xanthomonas citri* pv. *eucalyptorum* and had an ANI >95% with reference strains of *X. citri* pv. *citri* and *X. citri* pv. *phaseoli*.

This whole-genome shotgun project has been deposited at DDBJ/EMBL/GenBank under accession no. JAZGJM000000000. Annotation of the assembled genomes was done using the IMG Annotation Pipeline (v5.0.23) at the U.S. Department of Energy (DOE) Joint Genome Institute (JGI) (https://img.jgi.doe.gov) under accession no. Gp0621574.

## Abbreviations

ANI: average nucleotide identity
DDH: DNA–DNA hybridization
NA: nutrient agar
*Xae*: *Xanthomonas axonopodis* pv. *eucalyptorum*
*Xce*: *Xanthomonas citri* pv. *eucalyptorum*

## References

[1] Bophela KN, Venter SN, Stephanus N, Wingfield MJ, Duran A (2019). Xanthomonas perforans: a tomato and pepper pathogen associated with bacterial blight and dieback of Eucalyptus pellita seedlings in Indonesia. http://hdl.handle.net/2263/76946.

[2] Ferraz HGM, Badel JL, da Silva Guimarães LM, Reis BP, Tótola MR (2018). *Xanthomonas axonopodis* pv. eucalyptorum pv. nov. causing bacterial leaf blight on Eucalypt in Brazil. Plant Pathol J.

[3] Choudhary M, Minsavage GV, Goss EM, Timilsina S, Coutinho TA (2024). Whole-genome-sequence-based classification of *Xanthomonas euvesicatoria* pv. *eucalypti* and computational analysis of the type III secretion system. Phytopathology.

[4] Subedi A, Kara S, Aysan Y, Minsavage GV, Timilsina S (2023). Draft genome sequences of 11 *Xanthomonas* strains associated with bacterial spot disease in Turkey. Access Microbiol.

[5] Bolger AM, Lohse M, Usadel B (2014). Trimmomatic: a flexible trimmer for Illumina sequence data. Bioinformatics.

[6] Bankevich A, Nurk S, Antipov D, Gurevich AA, Dvorkin M (2012). SPAdes: a new genome assembly algorithm and its applications to single-cell sequencing. J Comput Biol.

[7] Seemann T (2014). Prokka: rapid prokaryotic genome annotation. Bioinformatics.

[8] Pritchard L, Glover RH, Humphris S, Elphinstone JG, Toth IK (2016). Genomics and taxonomy in diagnostics for food security: soft-rotting enterobacterial plant pathogens. Anal Methods.

[9] Richter M, Rosselló-Móra R (2009). Shifting the genomic gold standard for the prokaryotic species definition. Proc Natl Acad Sci U S A.

[10] Meier-Kolthoff JP, Carbasse JS, Peinado-Olarte RL, Göker M (2022). TYGS and LPSN: a database tandem for fast and reliable genome-based classification and nomenclature of prokaryotes. Nucleic Acids Res.

